# Gut Microbiome Profiling of the Endangered Southern Greater Glider (*Petauroides volans*) after the 2019–2020 Australian Megafire

**DOI:** 10.3390/ani13223583

**Published:** 2023-11-20

**Authors:** Jordyn Clough, Sibylle Schwab, Katarina Mikac

**Affiliations:** 1 School of Earth, Atmospheric and Life Sciences, Faculty of Science, Medicine and Health, University of Wollongong, Wollongong, NSW 2522, Australia; kmikac@uow.edu.au; 2 School of Medical, Indigenous and Health Sciences, Faculty of Science, Medicine and Health, University of Wollongong, Wollongong, NSW 2522, Australia

**Keywords:** greater glider, southern greater glider, endangered, microbiome, arboreal mammal, marsupial, bushfire, health, biodiversity

## Abstract

**Simple Summary:**

The gut microbiome can provide valuable information on the ecology and health of wildlife. We profiled the gut microbiota of the southern greater glider, an endangered Australian marsupial that was heavily disturbed by the 2019–2020 Australian bushfire season. Geographic location was found to be a significant driver of gut microbial community diversity and structure. In addition, the wildfires were shown to shape some aspects of the gut microbiome. By establishing baseline microbiome data for the southern greater glider, we lay the foundation for the future monitoring of populations at risk of compromised health, as well as inform future conservation management decisions for this endangered species.

**Abstract:**

Studying the gut microbiome can provide valuable insights into animal health and inform the conservation management of threatened wildlife. Gut microbiota play important roles in regulating mammalian host physiology, including digestion, energy metabolism and immunity. Dysbiosis can impair such physiological processes and compromise host health, so it is essential that the gut microbiome be considered in conservation planning. The southern greater glider (*Petauroides volans*) is an endangered arboreal marsupial that faced widespread habitat fragmentation and population declines following the 2019–2020 Australian bushfire season. This study details baseline data on the gut microbiome of this species. The V3–V4 region of the 16S rRNA gene was amplified from scats collected from individuals inhabiting burnt and unburnt sites across southeastern Australia and sequenced to determine bacterial community composition. Southern greater glider gut microbiomes were characterised by high relative abundances of Firmicutes and Bacteroidota, which is consistent with that reported for other marsupial herbivores. Significant differences in gut microbial diversity and community structure were detected among individuals from different geographic locations. Certain microbiota and functional orthologues were also found to be significantly differentially abundant between locations. The role of wildfire in shaping southern greater glider gut microbiomes was shown, with some significant differences in the diversity and abundance of microbiota detected between burnt and unburnt sites. Overall, this study details the first data on greater glider (*Petauroides*) gut microbiomes, laying the foundation for future studies to further explore relationships between microbial community structure, environmental stressors and host health.

## 1. Introduction

The microbiome has been recognised as an increasingly important aspect of threatened wildlife health [1] and there is a strong need to consider species’ microbiomes when planning for effective conservation management. The gut microbiome refers to the community of microorganisms that live within the gastrointestinal tract of a host. Gut microbiota play important symbiotic roles in regulating host physiological processes [2], including immunity [3], metabolism [4] and behaviour [5]. Imbalance, or dysbiosis, of the gut microbiome can have detrimental effects on host health [6], so preserving a healthy, balanced gut microbiome is vital for maintaining host fitness. Environmental stressors can shape microbial community structure [7,8]. Stressful events can cause the release of stress hormones that regulate immunity in the gut, which can alter microbial composition [9]. Profiling the gut microbiome therefore has the potential to reveal the impacts of disturbance events, such as wildfires, on gut symbiosis.

Consideration of the microbiome is especially important for obligate dietary specialists, which often have specific microbial assemblages adapted for the effective digestion of certain food types [10]. Greater gliders (*Petauroides*) are specialist herbivores endemic to eastern Australia with a diet that consists almost exclusively of foliage from the genus *Eucalyptus* [11,12]. The southern greater glider (*Petauroides volans*) is one of three species of greater glider, with a reported distribution from Prosperine, QLD through NSW and the ACT into VIC [13]. In July 2022, the species was uplisted to endangered under the National Environment Protection and Biodiversity Conservation Act 1999, citing rapid population declines and critical habitat destruction after the 2019–2020 Australian bushfire season as evidence that the species is now a conservation priority [14]. The koala is another arboreal herbivore that shares a similar *Eucalyptus*-centric diet, for which the gut microbiome has been well characterised [15,16,17,18,19,20]. Dietary differences caused by differential consumption of the foliage of different *Eucalyptus* sp. have been found to profoundly influence the gut microbiome of koalas [18]. This could have important implications for translocating specialist *Eucalyptus* folivores, such as the southern greater glider. Differences in diet between the source and destination habitats may contribute to poor translocation and overall health outcomes, with the translocated animals lacking gut microbiomes adapted to the new diet. To reduce this risk, careful consideration of the gut microbiome of the target population is necessary.

Microbial communities can be characterised by amplicon sequencing targeting the 16S ribosomal RNA gene, which is highly conserved in bacteria and remains the gold standard in microbial typing [21]. Molecular analyses of 16S have been a staple of microbial research for decades [22]. High-throughput, short-read sequencing of 16S based on the Illumina MiSeq platform is designed to specifically target the V3–V4 region and offers high taxonomic resolution at a reduced cost compared to other next-generation sequencing platforms [23]. In the context of Australian marsupials, previous work has focused on using 16S sequencing to characterise gut microbiota of select members of the Dasyuridae [24,25,26], Vombatidae [17,27] and Phascolarctidae [15,16,17,18,20]. However, the gut microbiome has yet to be formally investigated through 16S sequencing in the Pseudocheiridae family, which includes all extant greater gliders and ringtail possums.

To characterise gut microbiota of the southern greater glider and explore relationships between fire and gut microbiome composition, we analysed scat samples from twenty-five individuals across seven locations in southeastern NSW, including a mix of habitats that were burnt and remained unburnt during the 2019–2020 Australian bushfire season. To our knowledge, this is the first report of the southern greater glider (*Petauroides volans*) microbiome and provides baseline gut microbial community data for this species. Successive studies will benefit from having benchmark microbiome data available for greater gliders and the wider Pseudocheiridae family, laying the foundation for future studies to further explore relationships between microbial community structure, environmental stressors and host health.

## 2. Materials and Methods

### 2.1. Study Area

**Figure 1:**
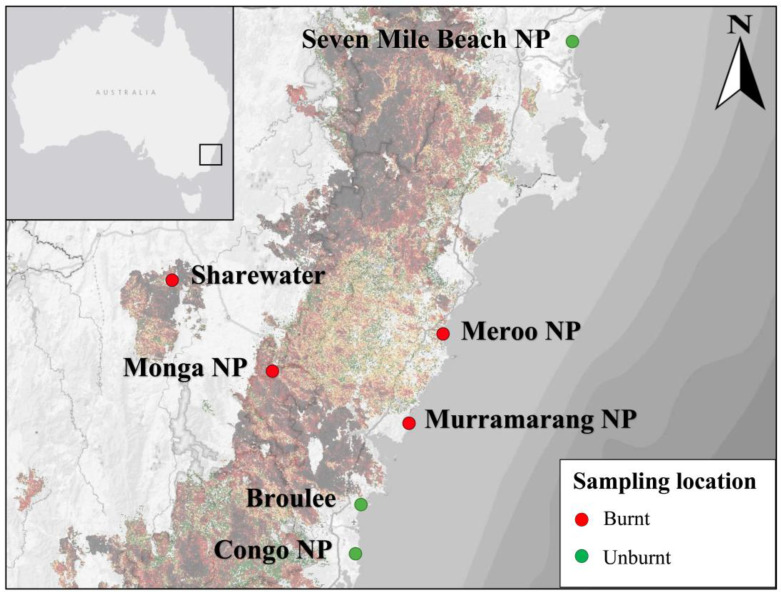
Map of southern greater glider sampling locations across southeastern NSW, Australia. Locations include Seven Mile Beach National Park (NP); Meroo NP; Murramarang NP; a public reserve in Broulee; Eurobodalla NP; Monga NP; and Sharewater, a wildlife sanctuary near Braidwood. Fire severity was taken from NSW Department of Planning and Environment Fire Extent and Severity Mapping (FESM) 2019/20 overlay [29]. Sampling locations are marked as burnt (red) or unburnt (green). Map generated using qGIS v3.32.1.

Scats were collected from twenty-five southern greater gliders (*n* = 11 male, *n* = 14 female) at seven sites across southeastern NSW in late 2021 (Figure 1). Localities included Seven Mile Beach National Park (NP) (*n* = 4), Broulee (*n* = 4), Eurobodalla NP (*n* = 3), Meroo NP (*n* = 3), Monga NP (*n* = 6), Murramarang NP (*n* = 3) and the wildlife sanctuary Sharewater (*n* = 2). Sites sampled were burnt to varying degrees during the 2019–2020 Australian bushfire season, with forest canopies ranging from unburnt to completely burnt (detailed in [28]). Dominant vegetation classes, reported *Eucalyptus* species, temperature ranges, annual mean rainfall, burn status during the 2019–2020 bushfire season and estimated greater glider effective population sizes for each location are provided in Table A1.

### 2.2. Sample Collection

Scats were collected from individuals following live capture from tree hollows (as described in [30]) and stored dry in sterilised vials at −20 °C prior to microbial analyses. All scats were collected directly from the cloaca upon discharge to prevent microbial contamination and ensure the samples could be correctly matched to the individual. In the laboratory, 250 mg faecal material from each individual was weighed, transferred to a sterilised safe-lock microtube (Eppendorf, Hamburg, Germany) and sent to the Australian Genome Research Facility (AGRF, Adelaide, Australia) on dry ice for DNA extraction and 16S microbial profiling. All research protocols were approved by the University of Wollongong Animal Ethics committee (AE19/02) and conducted under an NSW DPIE Scientific Licence (SL101968).

### 2.3. DNA Extraction and 16S Microbial Diversity Profiling

DNA extraction was performed by the AGRF as part of their Microbiome DNA Extraction Service. For 16S microbial profiling, the AGRF applied a polymerase chain reaction (PCR) to amplify the 300 bp V3–V4 341F-806R region of the 16S rRNA gene using validated forward primer (5′-CCTAYGGGRBGCASCAG-3′) and reverse primer (5′-GGACTACNNGGGTATCTAAT-3′) sequences. Amplicons were pooled and sequenced on the Illumina MiSeq platform using Nextera XT Indexes and paired-end sequencing chemistry, generating paired-end .fastq sequences. Demultiplexing, quality control and amplicon sequence variant (ASV) calling was then performed. Reads were analysed using QIIME 2 2019.7 [31]. Demultiplexed raw reads were primer-trimmed and quality-filtered using the cutadapt plugin, followed by denoising with DADA2 [32]. Taxonomy was assigned to ASVs in QIIME v2023.2 using the q2-feature-classifier classify-sklearn Naive Bayes taxonomy classifier [33] trained on the SILVA 138.1 small subunit rRNA database [34] with a 97% confidence threshold.

### 2.4. Microbial Diversity Analyses

Alpha diversity and beta diversity of gut microbial communities were analysed in QIIME2 v2023.2 [31]. Alpha diversity measures the diversity of microbial taxa within individual samples, while beta diversity measures the diversity of microbial community composition among samples. Alpha diversity was assessed by calculating the observed number of ASVs [35], Chao1 index [36] and Shannon index [37]. Boxplots for each alpha diversity metric were generated in R v4.3.1 using the ggplots2 package. Kruskal–Wallis tests were used to test for statistically significant (*p* < 0.05) differences in alpha diversity among samples based on location, burn status of the habitat during the 2019–2020 bushfire season, sex and month of scat collection. Amplicon sequence variant richness was determined by calculating the mean number of ASVs +/− the standard error of the mean for each location. Beta diversity was assessed by calculating unweighted UniFrac distances [38], weighted UniFrac distances [39], Bray–Curtis dissimilarity [40] and Jaccard distances [41]. Matrices of the distances between pairs of samples were generated for each beta diversity metric. Principal coordinate analysis (PCoA) of the matrices was conducted in QIIME2 v2023.2 and visualised by generating scatter plots in R v4.3.1 using the ggplots2 package. Permutational multivariate analysis of variance (PERMANOVA) tests, with 999 permutations, were used to test for statistically significant (*p* < 0.05) differences in beta diversity between samples.

### 2.5. Taxonomic Composition Profiling

Taxonomy bar plots were generated in R v4.3.1 using the qiime2R package. Taxa relative abundance (mean % +/− SEM) was compared at the phylum, family and genus levels. For baseline characterisation of the southern greater glider gut microbiome, the mean relative abundances of taxa and Firmicutes/Bacteroidota (F:B) ratio were calculated using data from twenty-four individuals (*n* = 24). It should be noted that one individual from Eurobodalla NP (CONGO40) was deemed to be an outlier due to disproportionately skewed abundances of dominant gut phyla (F:B ratio of 194.2:1). This individual was excluded from all analyses to determine accurate baseline microbial community information. To investigate differences in the dominant bacterial phyla between populations across southeastern NSW, variations in taxa abundance were compared (i) among geographic locations across southeastern NSW, and (ii) between burnt and unburnt sites. Wilcoxon rank sum tests were used to test for statistically significant (*p* < 0.05) differences in the relative abundances of taxa between groups.

### 2.6. Prediction of Functional Profiles of Microbial Communities

The Phylogenetic Investigation of Communities by Reconstruction of Unobserved States (PICRUSt2) program was used to predict metagenome functional profiles of southern greater glider gut microbial communities based on 16S sequencing data [42]. KEGG orthologue (KO) abundance output was analysed in R v4.3.1. KOs of interest were checked against the KO and KEGG databases to annotate individual genes and describe the functional pathways in which the identified microbial communities are implicated [43]. Wilcoxon rank sum tests were used to test for statistically significant (*p* < 0.05) differences in the relative abundance of KOs between populations from separate geographic locations.

### 2.7. Differential Abundance Analysis

Analysis of composition of microbiomes (ANCOM) was run in QIIME2 v2023.2 and used to test for significantly differentially abundant microbial taxa and KOs (i) among locations and (ii) between burnt and unburnt sites. This method of differential abundance analysis accounts for the compositional constraints of microbiome data by performing an additive log ratio transformation prior to comparing microbial community composition among samples [44]. In the ANCOM volcano plot output, the W value represents the number of times the null hypothesis (that the average abundance of a given taxon/KO in one population is equal to that in another population) is rejected for a given taxon/KO and the centred logarithmic ratio (clr) value indicates the effect size. 

### 2.8. Data Availability

All 16S rRNA gene sequence data generated within this study have been uploaded to the National Centre for Biotechnology Information (NCBI) open access Sequence Read Archive under the BioProject accession number PRJNA1026323.

## 3. Results

### 3.1. Microbial Diversity

**Figure 2:**
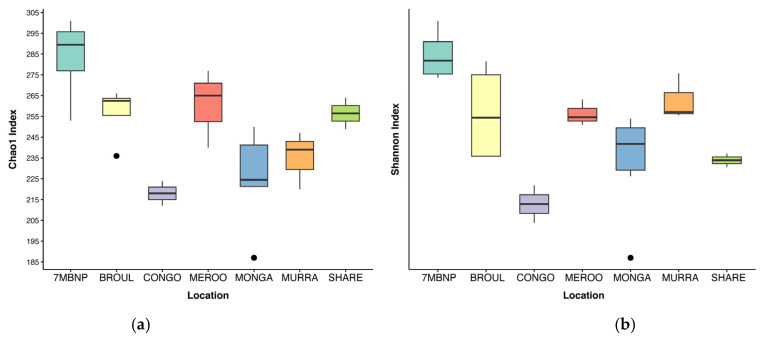
Alpha diversity of southern greater glider gut microbiomes from southeastern NSW. Boxplots show mean alpha diversity of samples at each location, with interquartile range and outlier values indicated. (**a**) Chao1 index. (**b**) Shannon index. 7MBNP = Seven Mile Beach NP (*n* = 4), BROUL = Broulee (*n* = 4), CONGO = Eurobodalla NP (*n* = 2), MEROO = Meroo NP (*n* = 3), MONGA = Monga NP (*n* = 6), MURRA = Murramarang NP (*n* = 3), SHARE = Sharewater (*n* = 2).

Alpha diversity was significantly different among geographic locations (Kruskal–Wallis test, *p* < 0.05) (Table 1). Gut microbial diversity was greatest in individuals from Seven Mile Beach NP and lowest in individuals from Eurobodalla NP (Figure 2). No significant differences in alpha diversity were detected between burnt and unburnt sites (Table 1). Additionally, alpha diversity did not significantly vary based on the sex of the greater glider or the month of scat collection (Table 1). A total of 1093 ASVs were identified across the 24 southern greater glider gut samples, with a mean of 244 ± 34 ASVs per sample. The greatest ASV richness was observed in individuals from Seven Mile Beach NP (283 ± 11 ASVs), while individuals from Eurobodalla NP had the lowest ASV richness (218 ± 6 ASVs). No significant differences in ASV richness were detected between burnt and unburnt sites.

**Figure 3:**
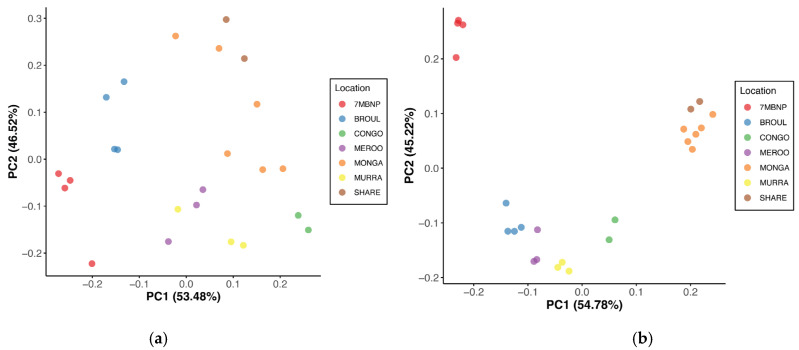
Principal coordinate analysis of beta diversity distances between southern greater glider gut microbiomes. (**a**) Bray–Curtis distances. (**b**) Jaccard distances. The proportion of variance among the samples explained by each axis is indicated in brackets. Individuals sampled from various geographic locations across southeastern NSW. 7MBNP = Seven Mile Beach NP (*n* = 4), BROUL = Broulee (*n* = 4), CONGO = Eurobodalla NP (*n* = 2), MEROO = Meroo NP (*n* = 3), MONGA = Monga NP (*n* = 6), MURRA = Murramarang NP (*n* = 3), SHARE = Sharewater (*n* = 2). Plots generated in R v4.3.1.

Geographic location was found to significantly influence gut microbial community structure, with dissimilarity in microbiome composition observed among locations (PERMANOVA, *p* < 0.05) (Table 2). Principal coordinate analysis of Bray–Curtis distances revealed strong clustering of samples based on location, where individuals from the same site had microbiomes that were more similar to each other than to individuals from disparate locations (Figure 3a). This was consistent with the PCoA of Jaccard distances, which similarly showed a strong clustering pattern based on location (Figure 3b). Clustering together of individuals from Monga NP and Sharewater was observed in both plots, as was the clustering of individuals from Meroo NP and Murramarang NP (Figure 3a,b). Individuals from Eurobodalla NP were also clearly separated from individuals from other locations (Figure 3a,b). Jaccard, Bray–Curtis and unweighted UniFrac distances significantly differed between burnt and unburnt sites (PERMANOVA, *p* < 0.05), although differences in weighted UniFrac distances were not significant (Table 2). No significant sex-based influence on gut microbial diversity was identified (Table 2).

### 3.2. Taxonomic Composition

Microbiome composition was investigated at the phylum, family and genus taxonomic levels to characterise dominant microbial groups in the gut of southern greater gliders.

#### 3.2.1. Phylum Level

**Figure 4:**
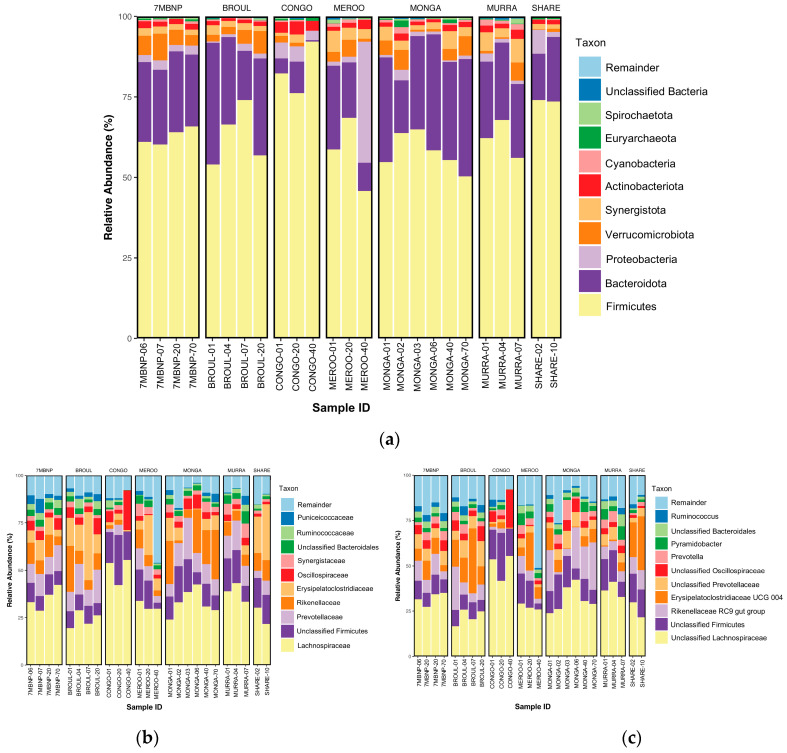
Taxonomic bar plots showing relative abundances of microbial taxa in southern greater glider gut microbiomes. (**a**) Phylum-level plot. (**b**) Family-level plot. (**c**) Genus-level plot. The size of the coloured regions on each plot indicates the proportional contributions (%) of each taxon to total composition (100%). Individuals sampled from various geographic locations across southeastern NSW. 7MBNP = Seven Mile Beach NP (*n* = 4), BRO = Broulee (*n* = 4), CONGO = Eurobodalla NP (*n* = 2), MEROO = Meroo NP (*n* = 3), MONGA = Monga NP (*n* = 6), MUR = Murramarang NP (*n* = 3), SW = Sharewater (*n* = 2). Plots generated in R v4.3.1.

Fourteen phyla were detected, with six phyla having a >1% relative abundance. Gut microbiomes were dominated by Firmicutes (mean relative abundance ± SEM: 63.12% ± 1.80%) and Bacteroidota (23.27% ± 1.79%), with a mean F:B ratio of 2.7:1. The next most abundant phyla were Proteobacteria (3.62% ± 1.51%), Verrucomicrobiota (3.31% ± 0.49%), Synergistota (2.98% ± 0.35%) and Actinobacteriota (1.80% ± 0.16%). Lower abundances (<1%) of Cyanobacteria, Spirochaeota, Desulfobacterota and Campilobacterota from the domain Bacteria, as well as Euryarchaeota and Thermoplasmatota from the domain Archaea, were also detected (Figure 4a).

#### 3.2.2. Family Level

Eighty-one families were detected, with 13 at >1% relative abundance. The Lachnospiraceae (33.04% ± 1.67%) were the dominant family in the southern greater glider gut, with the next five most abundant families being unclassified Firmicutes (12.28% ± 1.09%), Prevotellaceae (10.26% ± 0.94%), Rikenellaceae (9.82% ± 1.52%), Erysipelatoclostridiaceae (9.51% ± 1.86%) and Oscillospiraceae (3.70% ± 0.41%). Other families detected at lower relative abundances included Synergistaceae (2.98% ± 0.35%), unclassified Bacteroidales (2.84% ± 0.25%), Ruminococcaceae (2.11% ± 0.28%), Puniceicoccaceae (1.99% ± 0.37%) and Enterobacteriaceae (1.65% ± 0.70%) (Figure 4b).

#### 3.2.3. Genus Level

Ninety-nine genera were detected, but only 14 were found to be at >1% relative abundance. Among these, unclassified Lachnospiraceae had the greatest relative abundance (31.06% ± 1.69%), following unclassified Firmicutes (12.28% ± 1.09%), the *Rikenellaceae RC9 group* (9.73% ± 1.53%), *Erysipelatoclostridiaceae UCG 004* (9.50% ± 1.86), unclassified Prevotellaceae (5.74% ± 0.60%), unclassified Oscillospiraceae (3.66% ± 0.41%), *Prevotella* (3.69% ± 0.45%), *Pyramidobacter* (2.96% ± 0.35%), unclassified Bacteroidales (2.84% ± 0.25%), *Ruminococcus* (2.11% ± 0.28%), *Cerasicoccus* (1.99% ± 0.37%), *Shuttleworthia* (1.95% ± 0.41%) and *Escherichia-Shigella* (1.64% ± 0.69%) (Figure 4c).

To investigate differences in the dominant bacterial phyla among populations across southeastern NSW, the mean F:B ratio was calculated for each sampling location. The F:B ratio was significantly higher at Eurobodalla NP (12.7:1) compared to that observed at Sharewater (4.3:1), Meroo NP (3.1:1), Murramarang NP (2.6:1), Seven Mile Beach NP (2.6:1), Broulee (2.3:1) and Monga NP (1.9:1) (Kruskal–Wallis test, *p* < 0.05). It should be noted that the excluded outlier individual from the Eurobodalla NP population (CONGO40) had an F:B ratio of 194.2:1.

To observe the potential impacts of fire on gut microbiome composition, the data were then stratified based on whether the sample was collected from a site that was burnt during the 2019–2020 wildfires. At the phylum level, compositional similarity was indicated between samples collected from burnt and unburnt habitats, with no significant differences in the relative abundances of Firmicutes, Bacteroidota, Proteobacteria and Verrucomicrobiota. However, the relative abundance of Synergistota was found to be significantly greater in samples collected from burnt habitats (3.67%) compared to unburnt habitats (2.01%) (Wilcoxon rank sum test, S = 88, *p* < 0.05) (Table 3). At the family level, similar relative abundances of Lachnospiraceae, unclassified Firmicutes, Erysipelatoclostridiaceae, Prevotellaceae and Rikenellaceae were found (Table 3). Likewise, at the genus level, similar levels of unclassified Lachnospiraceae, unclassified Firmicutes, the *Rickenellaceae RC9 group*, *Erysipelatoclostridiaceae UCG04* and unclassified Prevotellaceae were detected (Table 3).

Some ASVs were exclusively found in burnt or unburnt habitats. Four-hundred twenty-six ASVs were exclusive to burnt sites, detected in at least one individual from a burnt site but not at unburnt sites. Conversely, 270 ASVs were detected in at least one individual from an unburnt site, but not at burnt sites. Thirty-nine ASVs were common among all individuals sampled from burnt sites; however, none of these were found only in burnt habitats, whereas 72 ASVs were common among individuals from unburnt sites, with 1 ASV (d1d1a5a618360d5a64a3e9fe0a39e394: unclassified Lachnospiraceae) found exclusively in unburnt habitats.

### 3.3. Functional Profiling of Microbial Communities

**Figure 5:**
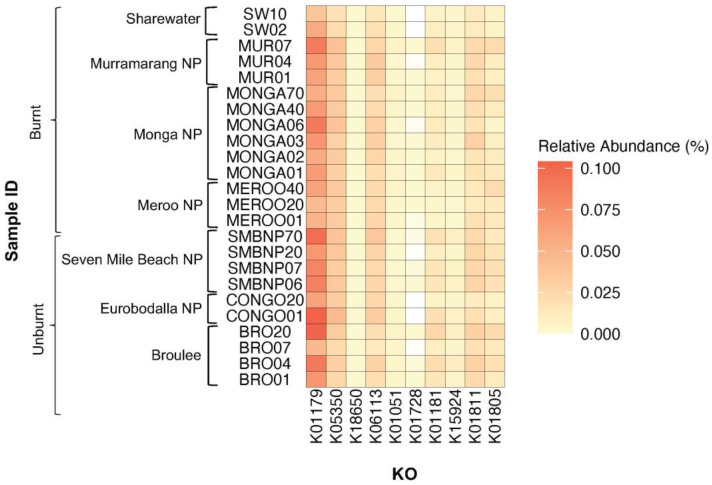
Heatmap plot showing relative abundance of KEGG orthologues (KOs) coding for plant-fibre-degrading enzymes detected across southern greater glider gut microbiomes from southeastern NSW. Geographic location and burn status of the sampling site are indicated. Relative abundance (%) of KO in each sample indicated by colour gradient. White cells indicate that the KO was not detected in the sample. K01179: endoglucanase, K05350: beta-glucosidase, K18650: exo-poly-alpha-galacturonosidase, K06113: arabinan endo-1,5-alpha-L-arabinosidas, K01051: pectinesterase, K01728: pectate lyase, K01181: endo-1,4-beta-xylanase, K15924: glucuronoarabinoxylan endo-1,4-beta-xylanase, K01811: alpha-D-xyloside xylohydrolase, K01805: xylose isomerase. Plot generated in R v4.3.1.

PICRUSt2 was used to predict functional profiles of 16S rRNA gene sequences sampled from southern greater glider scats. A total of 4630 KOs were common among all southern greater glider microbiomes, while 419 KOs were unique to individuals. Differences in the expression of KOs coding for plant-fibre-degrading enzymes were found among geographic locations (Figure 5, Table A3). The expression of K01181 (endo-1,4-beta-xylanase), K15924 (glucuronoarabinoxylan endo-1,4-beta-xylanase) and K01811 (alpha-D-xyloside xylohydrolase), coding for xylanases, was significantly different among locations (Wilcoxon rank sum test, *p* < 0.05). Likewise, the expression of K18650 (exo-poly-alphagalacturonosidase), coding for a pectinase, significantly differed among locations (Wilcoxon rank sum test, *p* < 0.05). No significant differences in the expression of KOs coding for cellulases were detected among locations.

Comparison between individuals from burnt and unburnt sites also revealed some differences in the expression of fibre-degrading KOs (Figure 5, Table A4). K01179 (endoglucanase) had a significantly higher abundance in the gut of individuals from unburnt sites (0.081%) compared to burnt sites (0.063%) (Wilcoxon rank sum test, S = 165, *p* < 0.05). Similarly, K15924 (Glucuronoarabinoxylan endo-1,4-beta-xylanase) had a significantly higher abundance at unburnt sites (0.0062% in unburnt vs. 0.0028% in burnt) (Wilcoxon rank sum test, S = 180, *p* < 0.05). In contrast, K01181 (endo-1,4-beta-xylanase) had a higher abundance at burnt sites (0.0095% in burnt vs. 0.016% in unburnt) (Wilcoxon rank sum test, S = 171, *p* < 0.05).

### 3.4. Differential Abundance Analysis

Differential abundance analysis was performed using ANCOM to identify significantly differentially abundant microbial taxa between southern greater glider gut microbiomes. The Defluviitaleaceae family was found to be differentially abundant between locations (W = 40). Defluviitaleaceae had the greatest relative abundance in individuals from Seven Mile Beach NP (0.06%), followed by Murramarang NP (0.05%), Broulee (0.05%), Monga NP (0.03%), Meroo NP (0.01%) and Eurobodalla NP (0.01%), while the family was not detected in individuals from Sharewater. The *Oscillospiraceae V9D2013 group* genus was also found to be differentially abundant between locations (W = 93). The relative abundance of the *Oscillospiraceae V9D2013 group* was highest in individuals from Seven Mile Beach NP (1.40%), with similar abundances at Murramarang NP (0.06%) and Broulee (0.06%), and a very low abundance at Meroo NP (<0.01%), while the genus was not detected at Eurobodalla NP, Monga NP or Sharewater. Twenty-two ASVs were found to be differentially abundant between geographic locations (Table A2) and evidence of site-specific microbial taxa was found. An unclassified Firmicutes, Bacteroidales, Prevotellaceae and two *Cerasicoccus* ASVs were detected only in the Seven Mile Beach NP population. Likewise, unclassified Lachnospiraceae ASVs were only found in the Eurobodalla NP and Monga NP populations, while an unclassified *Ruminococcus* ASV was only found in the Broulee population. An uncultured *Enterorhabdus* ASV was found among all South Coast populations (Seven Mile Beach NP, Broulee, Eurobodalla NP, Meroo NP, Murramarang NP), but not populations further inland (Monga NP, Sharewater). Additionally, an unclassified Lachnospiraceae ASV was found only in the inland populations. No differentially abundant phyla, families or genera were detected between burnt and unburnt sites.

**Figure 6:**
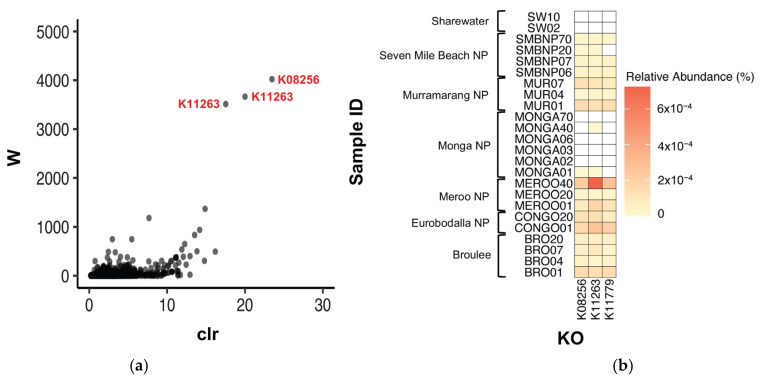
Differentially abundant KOs detected between southern greater glider gut microbiomes from across southeastern NSW. (**a**) Volcano plot from ANCOM differential abundance analysis. Only significantly differentially abundant KOs (assuming a 0.05 significance threshold) are labelled. The y-axis value (W) represents the number of times the null hypothesis (that the average abundance of a given KO in one population is equal to that in another population) was rejected. The x-axis value (clr) represents the effect size change between the compared populations. (**b**) Heatmap showing relative abundance of significantly differentially abundant KOs at each sampling location. Relative abundance (%) of KO in each sample indicated by colour gradient. White cells indicate that the KO was not detected in the sample. Plots generated in R v4.3.1.

ANCOM was also used to test for significantly differentially abundant KOs between southern greater glider gut microbiomes. Three KOs were found to be differentially abundant between locations, K08256 (W = 4022), K11779 (W = 3514) and K11263 (W = 3665) (Figure 6a). K08256 had the greatest relative abundance in individuals from Meroo NP (0.00016%) and Eurobodalla NP (0.00015%), followed by Murramarang NP (0.00011%), Broulee (0.00010%) and Seven Mile Beach NP (0.00003%). The KO was also detected at very low abundance in one individual from Monga NP (<0.00001%) but was absent in individuals from Sharewater (Figure 6b, Table A5). Following a similar pattern, K11779 also had greater relative abundances at Meroo NP (0.00016%) and Eurobodalla NP (0.00015%), with lower abundances detected in Murramarang NP (0.00011%), Broulee (0.00010%) and Seven Mile Beach NP (0.00003%), although the KO was absent from individuals from Monga NP and Sharewater (Figure 6b, Table A5). Finally, K11263 had the greatest relative abundance in individuals from Meroo NP (0.00031%), followed by Eurobodalla NP (0.00015%), Murramarang NP (0.00011%) and Broulee (0.00011%). Lower abundances were also detected at Seven Mile Beach NP (0.00003%) and Monga NP (<0.00001%), but as with the aforementioned KOs, K11263 was absent in individuals from Sharewater (Figure 6b, Table A5). No differentially abundant KOs were detected between burnt and unburnt sites.

## 4. Discussion

This study is the first characterisation of the southern greater glider gut microbiome and provides preliminary insights into the microbiome of greater gliders (*Petauroides*). Greater gliders are specialist folivores that exclusively rely on the leaves of *Eucalyptus* trees for nutrition [45]. Understanding the gut microbiome is of high importance when considering conservation actions for a dietary specialist species, as they often require specific microbial assemblages to effectively digest and harvest energy from their preferred food sources [19]. Analysis of the gut microbiome can also inform us of the health of individuals by identifying opportunistic pathogens [44,46] or dysbiosis, which can be a diagnostic of compromised health [47,48,49]. High localised extinction risks are predicted for many southern greater glider populations across southern NSW [28]. However, essential baseline health data are currently lacking for greater gliders, limiting the ability to make informed conservation management decisions for the species. Our study aimed to fill this gap in knowledge by characterising the gut microbiome of southern greater gliders from various geographic locations across southeastern NSW post-fire.

### 4.1. Southern Greater Glider Gut Microbiomes Exhibit Varied Microbial Diversity across the Landscape

Gut microbial diversity was found to significantly vary between southern greater gliders from different geographic locations, indicating that the distribution of microbial species in the gut is similar between individuals within a population but distinct between populations. This is supported by the clustering based on location observed in the PCoA of Bray–Curtis and Jaccard distance matrices (Figure 3), suggesting local habitat specificity in the gut microbial communities of southern greater gliders. Gut microbial diversity has been found to be substantially impacted by geographic location in a range of mammals [50,51,52]. Diet has been noted as a key factor in explaining differences in gut microbial diversity between populations [19,53,54,55,56]. The digestive systems of mammalian herbivores heavily rely on gut microbiota to assist in the extraction of nutrients and energy from their diet [57]. More diverse gut microbiomes have previously been associated with diets higher in fibre, while less diverse microbiomes have been linked to diets with less fibre and more starch content [58,59,60].

The higher microbial diversity observed at sampling locations dominated by South Coast Sands Dry Sclerophyll Forest (Seven Mile Beach NP, Broulee) could suggest that these populations are better adapted to the digestion of *Eucalyptus* sp. with high foliar fibre content. This is supported by a study which found that koalas feeding on the foliage of messmate stringybark (*Eucalyptus obliqua*), a high-fibre eucalypt of low nutritional quality, had gut microbiomes with significantly higher microbial diversity than koalas feeding on manna gum (*Eucalyptus viminalis*), which is lower in fibre and more nutritious [18]. South Coast Sands Dry Sclerophyll Forests are dominated by blackbutt (*Eucalyptus pilularis*) and bangalay (*Eucalyptus botryoides)* [61], with greater gliders found to have a preference for foraging the former at Seven Mile Beach NP [62]. Although no information is available on the foliar fibre content of blackbutt and bangalay at present, it is possible that these eucalypts are fibre-rich, and this has driven gut microbiomes to become more diverse. The significant differences in gut microbial diversity observed between locations in our study could therefore reflect the adaptation of gut microbiomes to locally available diets. Meanwhile, the sex of the animal and differences in sampling month had negligible impacts on gut microbial diversity and do not appear to be major drivers of southern greater glider gut microbiota.

Interestingly, individuals from Eurobodalla NP had microbiomes with substantially lower microbial diversity and were clustered together away from all other samples in the PCoA of Bray–Curtis and Jaccard distances (Figure 3). Collectively, these results suggest that there may be underlying factors at Eurobodalla NP that are influencing the gut microbial community structure of the resident southern greater glider population, whether that be environmental, genetic or a combination of both. Anthropogenic disturbances can induce dysbiosis of wildlife gut microbiomes [63] and could offer a potential explanation for the lack of microbial diversity observed at Eurobodalla NP. Recent work has shown that anthropogenic habitat disturbances can significantly disrupt the gut microbiome and lead to reduced microbial diversity in the gray-brown mouse lemur [64], Kuhl’s pipistrelle bat [65], Tome’s spiny rat and common opossum [66]. Eurobodalla NP is bounded by Moruya River to the north, Coila Lake to the south, and the Princes Highway and cleared land to the west, which act as major barriers to dispersal for arboreal marsupials. Human encroachment and loss of the surrounding old growth forest have resulted in the isolation and decline of the Eurobodalla NP greater glider population, culminating in its listing as an endangered population by the NSW State Government [67]. A recent study by Knipler et al. found that the Eurobodalla NP population exhibited the lowest effective population size of fourteen populations sampled across southeastern NSW [28]. In theory, microbial transmission between individuals via social interactions should be closely linked to population density. Social interactions have been shown to promote species richness within microbiomes in a variety of taxa [68,69,70]. Although considered generally solitary [71], social interactions between greater gliders have been documented [11] and population density is believed to shape some aspects of the species’ social organisation [72]. The reduced size of the Eurobodalla NP population, and consequently the decreased likelihood of social interactions between individuals, could thus play a role in preventing the assembly of diverse gut microbiomes.

### 4.2. Southern Greater Glider Gut Microbiomes Are Taxonomically Diverse

The dominance of Firmicutes and Bacteroidota in the gut of southern greater gliders is consistent with that reported in other marsupial herbivores, including the koala [17], common wombat [17], southern hairy-nosed wombat [27] and various macropods [73]. Like the koala, greater gliders are specialist folivores known to feed almost exclusively on *Eucalyptus* leaves [45]. Before the cell contents of *Eucalyptus* foliage can be digested and used as an energy source, the cell walls must be broken down. Previous studies have shown that the efficiency of digesting the cell walls of *Eucalyptus* leaf cells is low among arboreal marsupial herbivores [74,75,76], citing their high fibre content as a major challenge for digestion. The acquisition of fibre-degrading bacteria in the gut is therefore crucial to enable the host to efficiently digest and extract energy from a high-fibre *Eucalyptus* diet. Both the koala and greater gliders are hindgut fermenters, with a small, simple stomach and enlarged cecum that acts as the primary site of microbial activity [74,76]. A similarity in the overall taxonomic composition of the gut microbiomes of these species was therefore expected.

The F:B ratio is widely accepted to have an important influence on intestinal homeostasis [77] and is often discussed in the context of host health. Changes in this ratio have been implicated in various pathologies in animals [78,79,80]. An increase in F:B ratio has been associated with increased energy harvest and a heightened risk of obesity, while a decrease in F:B ratio has been linked to weight loss [78,81,82]. In our study, we established a baseline F:B ratio for the southern greater glider (2.7:1) by sampling individuals across a wide geographic spread in southeastern NSW. While the F:B ratio was observed to be significantly higher at Eurobodalla NP, the relative proportions of these dominant phyla in the gut microbiome remained similar across the other geographic locations. Having baseline F:B ratio data established for the southern greater glider will benefit future studies by enabling intra- and inter-species comparisons of dominant phyla.

The monitoring of this ratio may also be useful in assessing the health status of individuals or populations, with deviance from the baseline F:B ratio a potential biomarker for compromised health. For instance, the exceedingly high F:B ratio observed for the outlier individual sampled from Eurobodalla NP (194.2:1) is indicative of extreme dysbiosis and could suggest that this animal is suffering from a pathology that is critically impacting their gut ecosystem. While not to the same degree of extremity, a high F:B ratio was also observed for another individual sampled from Eurobodalla NP (12.7:1) relative to the greater gliders from other locations. This, coupled with the low gut microbial diversity observed in samples collected from Eurobodalla NP, appears to be indicative of an underlying problem in either the habitat or population itself that is causing an overdominance of Firmicutes and suppression of Bacteroidota in the gut. It is important to note that, while the F:B ratio can be informative and is commonly reported in microbiome studies of wildlife, dysbiotic increases or decreases in other phyla are not necessarily captured by this ratio. For example, sustained increases in the abundance of the phylum Proteobacteria have been found to contribute to dysbiosis [83], so the F:B ratio cannot be used on its own as a tell-all biomarker for gut health.

The dominance of the Lachnospiraceae family observed in the southern greater glider gut was also expected, as this family is a major constituent of mammalian gut microbiomes [84]. The Lachnospiraceae have previously been shown to be abundant in the gut microbiomes of other marsupials, such as the koala [17,19], common opossum and gray four-eyed opossum [66]. The Lachnospiraceae encode key enzymes involved in the breakdown of carbohydrates, sugar transport mechanisms and metabolic pathways that are highly specialised for the degradation of complex plant material [85]. The family is known to be fibrolytic and can breakdown plant fibres, such as cellulose and hemicellulose, that are otherwise indigestible by the host, enabling them to be harvested and used for energy [85]. Other abundant families included the Prevotellaceae, Rikenellaceae and Oscillospiraceae. The Prevotellaceae contains species with extensive polysaccharide and protein degradation capabilities [86,87,88]. Meanwhile, the Rikenellaceae and Oscillospiraceae are two of the most dominant bacterial families found in herbivore microbiomes [89]. The former has been shown to be highly abundant in the gut of the southern hairy-nosed wombat [27] and various ruminants [89], as well as being enriched in mice fed a high-fat diet [90]. The latter contains species that play crucial roles in cellulose degradation [91] and are dominant in the gut of ruminants such as giraffes, cattle and hartebeest [92].

The high relative abundances of the *Rikenellaceae RC9 group* and *Erysipelatoclostridiaceae UCG 004* observed in our study further support the notion that the southern greater glider microbiome is well adapted to the degradation of a fibre-rich plant diet. A positive relationship between *Rikenellaceae RC9 group* abundance and dietary fibre has been previously confirmed in cattle [93,94]. Likewise, the *Erysipelatoclostridiaceae UCG 004*, which contains fermentative and fatty-acid-synthesising bacteria [95], has been shown to be a core genus occurring in the gut of healthy newborn calves [96]. Surprisingly, the *Ruminococcus* genus, which contains important cellulolytic bacteria [57] that dominate the koala gut [16], was detected in low abundances in southern greater glider gut microbiomes. Despite their similar *Eucalyptus* diets, it appears that either the *Ruminococcus* play less prominent roles in fibre digestion in greater gliders compared to koalas or that, despite making up a smaller proportion of the gut microbiome, they remain a key contributor to fibrolysis.

We observed patterns of differential abundance among ASVs that underscore the presence of site-specific microbial taxa, further supporting an intrinsic link between the gut microbiome and local environment. We identified twenty-two ASVs that showed differential abundance between geographic locations, encompassing diverse taxonomic groups such as Firmicutes, Lachnospiraceae, Bacteroidales, Burkholderiales, *Enterorhabdus*, *Cerasicoccus* and *Ruminococcus*. Some ASVs were exclusive to populations, suggesting the accumulation of specific microbiota to suit local conditions. For instance, unclassified Firmicutes, Bacteroidales, Prevotellaceae and *Cerasicoccus* ASVs were solely found in the Seven Mile Beach NP population, reflecting the high gut microbial diversity observed. These findings highlight the importance of local environmental factors in shaping the microbiome in different geographic contexts. Additionally, the presence of unique *Enterorhabdus* and Lachnospiraceae ASVs found only in coastal and inland populations, respectively, suggests potential microbiome adaptations driven by ecological or climatic factors that vary with proximity to the coast. It has been shown that gut microbiota can vary between high- and low-altitude environments in various mammals, including the domestic pig [97], wild plateau pika [98] and rhesus macaque [99]. Differences in elevation may therefore contribute to the selection of *Enterorhabdus* species in the gut of greater gliders from coastal (lower elevation) populations versus those found further inland (at higher elevation). Further research is needed to explore the functional significance of these site-specific microbial taxa, the factors driving their selection, and their potential impacts on greater glider ecology and health.

It should be acknowledged that the use of 16S rRNA amplicon sequencing limits the taxonomic resolution achievable in this study and overlooks taxa outside the domains Bacteria and Archaea. This could be improved by using whole-genome shotgun metagenomic sequencing [100], which enables the strain-level identification of taxa and can facilitate the detection of other microorganisms present in the gut, albeit at higher cost. It should also be noted that using faecal samples as a proxy for the gut has its own limitations in microbiome research. Previous work in koalas has shown that rectal swabs contain all of the diversity present in faecal samples along with additional taxa [16], suggesting that faecal microbial communities may only subsample overall gut microbial diversity. Nevertheless, the non-invasive nature of faecal sampling offers an effective way of profiling the gut microbiome of wild animals, while minimising the stress and risks of harm associated with more invasive sampling approaches.

### 4.3. Gut Microbial Community Functional Profiles Provide Insights into Greater Glider Metabolism, Diet and Health

Variability in the abundance of functional orthologues coding for fibre-degrading enzymes found across geographic locations further reflects dietary differences across the landscape. In particular, xylanases were found to show the most macrospatial variability, while no differences in the expression of cellulases were detected among locations. The enrichment of microbiota expressing endo-1,4-beta-xylanase in the gut of individuals from burnt sites could have important ecological and physiological implications. This enzyme catalyses the hydrolysis of xylans, complex hemicellulose carbohydrates found in plant cell walls, particularly in woody plant tissue [101]. In the aftermath of fire, there might be an increase in the availability of charred or partially burnt woody plant material to opportunistically forage. The enrichment of endo-1,4-beta-xylanase in individuals from burnt habitats may therefore provide a competitive advantage in a post-fire environment by enabling them to more effectively digest burnt plant material. Meanwhile, the lowered expression of endoglucanase in gliders from burnt sites could suggest that individuals in burnt habitats are not relying as heavily on cellulose-rich plant material in their diet. A previous study found that wild Asian elephant microbiomes had a greater abundance of hemicellulose-degrading enzymes than cellulases, with the authors’ suggesting that breaking down hemicelluloses over cellulose could maximise energy extraction from plant material [102]. Cellulose is generally a more recalcitrant and energy-intensive substrate to digest than hemicelluloses [103]. A community shift toward xylanase-producing microbiota may represent a strategy for greater gliders to conserve energy in a post-fire environment, where there are expected to be increased energy demands for foraging, mate-seeking and predator vigilance.

Of concern is the detection of the functional orthologue coding for phosphatidyl-myo-inositol alpha-mannosyltransferase in a number of coastal southern greater glider populations. Phosphatidyl-myo-inositol alpha-mannosyltransferase is an enzyme that is essential for the biosynthesis of the cell envelope of members of the *Mycobacterium tuberculosis* complex, which plays a key role in the pathogenesis of tuberculosis [104]. Tuberculosis is a global health threat, affecting wildlife [105], livestock [106] and domestic animals [107], as well as presenting significant zoonotic risk to humans [108]. Infection with *M. tuberculosis* bacteria can occur through inhalation, ingestion or dermal contact with the pathogen, presenting strong potential for both intraspecies and interspecies transmission [109]. The common brushtail possum is the primary wildlife reservoir of *M. bovis*, the causative agent of bovine tuberculosis, in New Zealand [110], although infection has never been reported in possums in Australia. While bovine tuberculosis was declared eradicated from Australia in 1997, infection control in the leadup was mainly limited to cattle, water buffalo and feral pigs, with no native wildlife reservoirs identified [111]. Due to its ability to infect multiple hosts and spread through varied transmission routes, it is plausible that, despite being eradicated in livestock, the *M. tuberculosis* complex could persist in Australian wildlife.

Detection of this orthologue in coastal greater glider populations could be a potential indicator that the species is acting as a reservoir of mycobacterial infection. Higher abundances of the orthologue were detected in the Meroo NP and Eurobodalla NP populations, which have previously been shown to have the lowest contemporary effective population sizes of all populations sampled on the NSW South Coast [28]. Due to their small size, it is possible that the populations at Meroo NP and Eurobodalla NP have experienced genetic drift and inbreeding effects [112,113,114] and this has contributed to reduced population genetic diversity [28]. Genetic diversity is crucial for a population’s ability to respond to disease outbreaks, with high genetic diversity increasing the chance of having individuals in the population carrying advantageous alleles that confer resistance or immunity to the pathogens. Reduced population genetic diversity could mean that the immune systems of the animals at Meroo NP and Eurobodalla NP are compromised due to a lack of immune-related adaptive genetic variability [115], making them more susceptible to infection. Meanwhile, in populations further inland inhabiting contiguous forest, and for which high levels of genetic diversity have been reported [28], the orthologue was detected only in trace amounts (Monga NP) or not at all (Sharewater). It should also be noted that the Meroo NP and Eurobodalla NP populations geographically occur in a current and historically important area for dairy production in southeastern NSW that has been ongoing since at least the mid-1800s [116], which might have increased risks of interspecies transmission prior to the eradication of tuberculosis from livestock. Moreover, the *M. tuberculosis* complex was recently detected in an Australian sea lion from South Australian waters in 2017 [117], marking the first report of tuberculosis in an Australian pinniped in over 20 years.

In response to these findings, we recommend that the populations identified to be at-risk are screened for mycobacterial infection as soon as practicable to mitigate the potential for an epizootic disease event.

### 4.4. Wildfire Affects the Presence and Abundance of Arboreal Marsupial Gut Microbiota

Our analyses revealed that the 2019–2020 wildfires had discernible impacts on southern greater glider gut microbial communities. While inhabiting a burnt habitat was found to have negligible influence on the levels of microbial diversity within the host’s microbiome, significant dissimilarity in microbial diversity was found between host populations from burnt and unburnt habitats estimated using Jaccard, Bray–Curtis and unweighted UniFrac metrics (Table 2). Although these results point to a significant relationship between microbial beta diversity and habitat burnt during the wildfires, when assessed using the weighted UniFrac metric, this was no longer found to be statistically significant. Weighted UniFrac accounts for the proportionality of the taxa shared between samples when calculating dissimilarity, whereas unweighted UniFrac focuses only on the presence or absence of taxa [38,39]. This suggests that while there are differences in the overall composition of gut microbial communities in greater glider populations from burnt versus unburnt habitats, when comparing the microbiomes based on the relative abundances of key taxa using weighted UniFrac, this distinction becomes less evident.

Gut microbiota are sensitive to environmental changes [8,118] and the 2019–2020 wildfires were a major disturbance event impacting ecosystems across southern and eastern Australia [119]. A recent study revealed that the 2019–2020 wildfires substantially altered the gut microbiome of Kangaroo Island echidnas [120]. The authors’ found no differences in alpha diversity between samples collected post fire from fire-affected and non-affected areas, which supports the findings of our study. Interestingly, they found that it was whether or not the sample was collected pre- or post-bushfire that drove significant changes in microbial community structure, with no clear differences between samples collected post fire from within or outside burnt areas [120]. This is incongruous with our results, which could be attributed to fundamental differences in the species biology or the severity of wildfire experienced on Kangaroo Island compared to southeastern NSW. Alternatively, the smaller sample size used in the aforementioned study (*n* = 7 post fire) may have introduced a stronger sampling bias compared to our study, which incorporated a considerably larger sample size (*n* = 25) and may thus provide a more accurate representation of the effects of fire on gut microbiomes. Habitat disturbances, more generally, have been well documented to impact gut microbial diversity in mammals [27,64,65,66,121,122]. For instance, gut microbial communities of the southern hairy-nosed wombat are more similar between individuals from degraded habitats than with those from undisturbed habitats [27]. This is consistent with the dissimilarity observed between the gut microbiomes of greater gliders from burnt versus unburnt habitats in our study.

Several taxonomic groups at the phylum, family and genus levels remained relatively stable between burnt and unburnt habitats, suggesting an overall resilience of gut microbiomes to wildfire disturbances. However, the Synergistota were found to be significantly enriched in greater glider microbiomes from burnt sites, suggesting that the phylum may play an important ecological role in post-fire conditions. The Synergistota as a phylum are poorly characterised. While some species have been shown to be incapable of breaking down carbohydrates, all Synergistota are able to ferment amino acids [123,124]. In post-fire habitats, where dietary carbohydrates may be scarce, the enrichment of Synergistota in the gut might provide a survival advantage by ensuring that amino acids can be efficiently fermented and used as an alternative energy source. Future research directed at investigating the functional properties of Synergistota in the southern greater glider gut would be valuable for better understanding how the species’ gut microbiome evolves in response to wildfire-mediated habitat disturbances.

The detection of ASVs unique to southern greater gliders from burnt and unburnt habitats further points to an influence of wildfire on the gut microbiome. Habitat degradation creates suboptimal habitats for wildlife, which can enforce dietary changes that result in a variable gut microbial community composition [121,122]. One study found that red colobus monkeys from degraded habitats lacked microbiota with the metabolic potential to detoxify plant xenobiotics, despite their presence in individuals from undisturbed habitats [122]. Likewise, others have found clear distinctions in the functional microbial communities of captive black rhinoceros [125], giant panda [126] and Nambian cheetah [127] compared to their wild counterparts. The reduction or loss of certain microbiota may reduce the functional capacity of the host’s gut microbiome, and for herbivorous mammals that rely on microbiota to facilitate the breakdown of plant material, losing the ability to process such food sources could have major implications for host survivorship. Resolving the taxonomic identity of the Lachnospiraceae ASV absent in the gut of individuals from burnt habitats could discern whether a lack of this microbial species has important functional implications for animals in a post-fire environment.

It should be acknowledged that variability in burn severity across a landscape can occur during large-scale wildfires due to habitat patches that have different vegetation types, moisture content and varying topography burning at different temperatures [128]. Patches experiencing lower-severity fires have been deemed an important source of greater glider refugia during wildfire events [129]. These refugia enable animals to survive wildfire and eventually repopulate the areas that experienced higher-severity fires [130]. This could have important implications on the gut microbiome of greater gliders. Fire refugia, where there are higher densities of greater gliders confined to a smaller area [129], could lead to increased contact between individuals. This could potentially facilitate the transfer of gut microbiota, symbiotic or pathogenic, from one animal to another [131]. While this might enhance microbiota transfer, and lead to beneficial or detrimental effects on the host, it does not necessarily guarantee increased microbiota diversity. In fact, the opposite may occur. When animals are confined to a smaller refuge area with limited food sources available, their diets may become more uniform. This could potentially lead to a loss of gut microbial diversity within the population, as the microbiomes of the survivors adapt to a shared, less diverse diet. An altered gut microbiome might, in turn, impact host fitness by affecting the animal’s ability to digest varied foods or adapt to environmental changes, which could influence their health, survival and reproductive success. Our baseline findings lay the foundation for future work by providing a reference point to assess the long-term consequences of post-fire changes in greater glider gut microbiota. Understanding the resilience of greater glider gut microbiomes to wildfire will be essential for improving our ability to predict and mitigate declines in population health, leading to better informed, more effective conservation management. 

## 5. Conclusions

Here, we report the first characterisation of the southern greater glider gut microbiome and provide baseline gut microbial data for *Petauroides*. This contributes to the limited, yet growing, body of knowledge on Australian marsupial microbiomes. We characterised predominant microbial taxa in the gut of southern greater gliders and found that geographic location had the greatest impact on gut microbial diversity and composition. Site-adapted taxa and functional profiles of microbial communities were evident, suggesting adaptation of the gut microbiome to suit the varying habitat conditions found across southeastern NSW. We also identified a potential indicator of pathogenic infection in coastal populations that warrants further investigation. The 2019–2020 wildfires were indicated to have some impact on the presence and abundance of specific microbiota in the southern greater glider gut, although the exact mechanism for this remains unclear. Future work should continue to focus on elucidating the role of wildfire in shaping the gut microbiomes of Australian arboreal marsupials, which will be of high value for conservation planning with predicted increases in the frequency and severity of wildfire events in Australia over the coming decades.

## Figures and Tables

**Table 1:** Alpha diversity of southern greater glider gut microbiomes. Individuals grouped based on sampling location, burn status of site, sex and month of collection. Analysis performed in QIIME2 v2023.2. Kruskal–Wallis test used to test for significant differences among groups. Test statistic (H) is indicated for each comparison. * = *p* < 0.05.

	Number of Observed Features (ASVs)	Chao1	Shannon
Location	15.52 *	15.52 *	16.18 *
Burn status of site	2.69	2.69	1.37
Sex	1.66	1.66	0.31
Month collected	8.35	8.35	5.28

**Table 2:** Beta diversity of southern greater glider gut microbiomes. Individuals grouped based on sampling location, burn status of site, and sex. Analysis performed in QIIME2 v2023.2. Permutational multivariate analysis of variance (PERMANOVA) used to test for significant differences between groups. Test statistic (pseudo F) is indicated for each comparison. * = *p* < 0.05.

	Bray–Curtis Distance	Jaccard Distance	Unweighted UniFrac Distance	Weighted UniFrac Distance
Location	3.64 *	3.33 *	2.14 *	2.86 *
Burn status of site	2.60 *	2.85 *	2.33 *	0.81
Sex	0.84	0.89	0.87	0.76

**Table 3:** Microbial taxa detected in southern greater glider gut microbiomes from burnt and unburnt sites across southeastern NSW. The five most dominant taxa at the phylum, family and genus taxonomic levels are shown. Relative abundance presented as mean (%) ± standard error of the mean (SEM). *p* values from Wilcoxon rank sum test indicated. * = *p* < 0.05.

Taxonomic Level	Taxa	Mean Relative Abundance (% ± SEM)		*p* Value
		Burnt Habitat	Unburnt Habitat	
Phylum	Firmicutes	61.01 ± 2.23	66.08 ± 8.96	0.23
	Bacteroidota	24.15 ± 2.21	22.03 ± 9.74	0.70
	Proteobacteria	4.45 ± 2.59	2.46 ± 1.47	0.33
	Verrucomicrobiota	2.75 ± 0.63	4.09 ± 2.38	0.15
	Synergistota	3.67 ± 0.52	2.01 ± 0.57	0.03 *
Family	Lachnospiraceae	32.78 ± 1.69	33.40 ± 3.35	0.70
	Unclassified Firmicutes	13.10 ± 1.31	11.15 ± 1.91	0.23
	Erysipelatoclostridiaceae	8.54 ± 2.36	10.87 ± 3.10	0.36
	Prevotellaceae	10.13 ± 1.24	10.45 ± 1.51	0.84
	Rikenellaceae	10.85 ± 2.18	8.38 ± 2.04	0.62
Genus	Unclassified Lachnospiraceae	30.98 ± 1.71	31.16 ± 3.41	0.77
	Unclassified Firmicutes	13.10 ± 1.31	11.15 ± 1.91	0.23
	*Rickenellaceae RC9 group*	10.77 ± 2.19	8.28 ± 2.04	0.62
	*Erysipelatoclostridiaceae UCG04*	8.54 ± 2.36	10.86 ± 9.81	0.36
	*Unclassified Prevotellaceae*	5.15 ± 0.57	6.57 ± 1.18	0.21

## Data Availability

The datasets generated and analysed during the current study are available under the BioProject accession number PRJNA1026323 from the National Center for Biotechnology Information Sequence Read Archive (https://dataview.ncbi.nlm.nih.gov/object/PRJNA1026323) (uploaded on 10 October 2023).

## Appendix A

**Table A1:** Southern greater glider sampling locations in southeastern NSW, Australia. Vegetation classes and reported *Eucalyptus* species sourced from the NSW State Vegetation Type Map [132]. Temperature ranges and rainfall data sourced from Bureau of Meterology using data from 2021 collected at the nearest Bureau station to the sampling location. Burn status of the habitat during the 2019–2020 Black Summer wildfires sourced from NSW Department of Planning and Environment Fire Extent and Severity Mapping (FESM) 2019/20 [29]. Estimates of effective population size taken from [28], where available.

Sampling Location	Dominant Vegetation Classes	Reported *Eucalyptus* Species	Monthly Mean Temperature Range for 2021 (°C)	Annual Mean Rainfall for 2021 (mm)	2019–2020 Fires Burn Status	Greater Glider Effective Population Size
Seven Mile Beach National Park, Gerroa	South Coast Sands Dry Sclerophyll Forests	*E. botryoides* *E. pilularis*	20.9	1580.6	Unburnt	45.1
South Broulee Beach, Broulee	South Coast Sands Dry Sclerophyll Forests	*E. botryoides* *E. pilularis*	20.1	1189.4	Unburnt	87.7
Eurobodalla National Park, Congo	Southern Lowland Wet Sclerophyll Forests	*E. pilularis* *E. botyroides* *E. scias* *E. agglomerata* *E. paniculata*	20.9	1144.9	Unburnt	6.2
Meroo National Park, Meroo	Southern Lowland Wet Sclerophyll Forests; Southeast Dry Sclerophyll Forests	*E. pilularis* *E. botyroides* *E. scias* *E. agglomerata* *E. paniculata* *E. agglomerata* *E. globoidea* *E. sieberi* *E. consididenia*	22.6	969.2	Burnt	8.2
Monga National Park, Monga	Southern Escarpment Wet Sclerophyll Forests	*E. cypellocarpa* *E. fastigata* *E. obliqua* *E. viminalis*	18.4	1473.0	Burnt	160.8
Murramarang National Park, Murramurang	Southeast Dry Sclerophyll Forests	*E. agglomerata* *E. paniculata* *E. agglomerata* *E. globoidea* *E. sieberi* *E. consididenia*	22.0	1092.4	Burnt	7.8
Sharewater, Tallaganda	Southern Escarpment Wet Sclerophyll Forests; Southeast Dry Sclerophyll Forests	*E. cypellocarpa* *E. fastigata* *E. obliqua* *E. viminalis* *E. agglomerata* *E. paniculata* *E. agglomerata* *E. globoidea* *E. sieberi* *E. blaxlandii* *E. dives* *E. smithii*	18.4	1261.4	Burnt	-

**Table A2:** Differentially abundant amplicon sequence variants (ASVs) detected between southern greater glider gut microbiomes across southeastern NSW. Amplicon sequence variants and the corresponding taxonomic classifications listed. ANCOM was used to determine significantly differentially abundant ASVs between sampling locations. W statistic represents the number of times the null hypothesis (the average abundance of an ASV in one location is equal to that in another location) was rejected for a given ASV, under a 0.05 significance threshold. Only significantly differentially abundant ASVs are listed. Relative abundance (mean % ± SEM) given for locations where the respective ASV was detected in at least one sample.

ASV	Taxonomic Classification	W	Mean Relative Abundance (% ± SEM)	
**27a92e9d957ed8ff16c0dfac8033bdf5**	d__Bacteria; p__Firmicutes; c__Clostridia; o__Lachnospirales; f__Lachnospiraceae	1091	Sharewater	2.11 ± 0.07
			Monga NP	1.71 ± 0.18
**24c0bed7fe8e9346c4a624644cf979e8**	d__Bacteria; p__Firmicutes; c__Clostridia; o__Lachnospirales; f__Lachnospiraceae	1091	Murramarang NP	2.59 ± 0.99
			Meroo NP	0.97 ± 0.37
**d1d1a5a618360d5a64a3e9fe0a39e394**	d__Bacteria; p__Firmicutes; c__Clostridia; o__Lachnospirales; f__Lachnospiraceae	1090	Broulee	0.81 ± 0.22
			Eurobodalla NP	0.80 ± 0.12
			Seven Mile Beach NP	0.60 ± 0.16
**bc2343861ddbca17c55ee68a21fe8e3a**	d__Bacteria; p__Firmicutes	1090	Seven Mile Beach NP	3.52 ± 0.20
**28b2ac3cac0b46d8b26a31f1ab59c922**	d__Bacteria; p__Actinobacteriota; c__Coriobacteriia; o__Coriobacteriales; f__Eggerthellaceae; g__Enterorhabdus; s__uncultured_bacterium	1084	Sharewater	0.41 ± 0.10
			Monga NP	0.38 ± 0.09
			Seven Mile Beach NP	0.24 ± 0.06
**c001095135fb918b7c1715ddecc36fa6**	d__Bacteria; p__Firmicutes; c__Clostridia; o__Lachnospirales; f__Lachnospiraceae	1082	Monga NP	0.51 ± 0.14
			Seven Mile Beach NP	0.28 ± 0.08
**8253eb9289121ce88046e7ebb4b642e5**	d__Bacteria; p__Actinobacteriota; c__Coriobacteriia; o__Coriobacteriales; f__Eggerthellaceae; g__Enterorhabdus; s__uncultured_bacterium	1071	Eurobodalla NP	0.99 ± 0.31
			Murramarang NP	0.64 ± 0.15
			Meroo NP	0.29 ± 0.09
			Seven Mile Beach NP	0.21 ± 0.03
			Broulee	0.17 ± 0.04
**f73e366c345a0d82a70413c5aec880b6**	d__Bacteria; p__Firmicutes; c__Clostridia; o__Lachnospirales; f__Lachnospiraceae	1009	Eurobodalla NP	0.74 ± 0.04
			Monga NP	0.89 ± 0.44
			Meroo NP	0.27 ± 0.07
			Murramarang NP	0.07 ± 0.03
**1bc20c352c5297c1e260d58aabeeb750**	d__Bacteria; p__Firmicutes; c__Clostridia; o__Lachnospirales; f__Lachnospiraceae	1033	Eurobodalla NP	5.04 ± 2.00
			Sharewater	2.30 ± 0.35
			Monga NP	1.57 ± 0.63
			Broulee	0.51 ± 0.18
			Meroo NP	0.34 ± 0.04
			Murramarang NP	0.33 ± 0.14
**e13ba7436b454d9525ce3e631b789355**	d__Bacteria; p__Verrucomicrobiota; c__Verrucomicrobiae; o__Opitutales; f__Puniceicoccaceae; g__Cerasicoccus; s__uncultured_bacterium	1065	Seven Mile Beach NP	4.02 ± 1.72
**bd537de474dbb1b14c5cc52cc017a309**	d__Bacteria; p__Bacteroidota; c__Bacteroidia; o__Bacteroidales	1046	Monga NP	0.39 ± 0.15
			Murramarang NP	0.34 ± 0.06
			Broulee	0.30 ± 0.05
			Eurobodalla NP	0.22 ± 0.17
			Sharewater	0.11 ± 0.03
**50f8d53a33780a64348ec66e38a33cdf**	d__Bacteria; p__Firmicutes; c__Clostridia; o__Lachnospirales; f__Lachnospiraceae	1026	Eurobodalla NP	1.95 ± 1.58
			Monga NP	0.33 ± 0.11
			Broulee	0.05 ± 0.01
			Murramarang NP	0.03 ± 0.03
**0786c15611bf814f2d41cf851a71b2b6**	d__Bacteria; p__Proteobacteria; c__Gammaproteobacteria; o__Burkholderiales	1048	Seven Mile Beach NP	1.35 ± 0.10
**b47ecf8a5559f8e193a4ca3cdbf074b6**	d__Bacteria; p__Firmicutes; c__Clostridia; o__Oscillospirales; f__Ruminococcaceae; g__Ruminococcus	1049	Broulee	1.67 ± 0.79
**021fe8edae51250b230154489e2dfb4c**	d__Bacteria; p__Bacteroidota; c__Bacteroidia; o__Bacteroidales; f__Prevotellaceae	1026	Seven Mile Beach NP	1.11 ± 0.28
**a75e2f333dcb0988db971ba7d81e950b**	d__Bacteria; p__Bacteroidota; c__Bacteroidia; o__Bacteroidales; f__Prevotellaceae	1013	Murramarang NP	0.38 ± 0.28
			Meroo NP	0.19 ± 0.05
			Broulee	0.10 ± 0.01
**41ef40105853acf4d6452e5e3c3859da**	d__Bacteria; p__Firmicutes; c__Clostridia; o__Lachnospirales; f__Lachnospiraceae	998	Murramarang NP	1.62 ± 0.24
			Meroo NP	1.53 ± 0.36
			Seven Mile Beach NP	0.43 ± 0.14
			Broulee	0.39 ± 0.08
			Eurobodalla NP	0.22 ± 0.03
			Sharewater	0.12 ± 0.12
**0656d5259d674955e7a3e2d1d511bc10**	d__Bacteria; p__Verrucomicrobiota; c__Verrucomicrobiae; o__Opitutales; f__Puniceicoccaceae; g__Cerasicoccus; s__uncultured_bacterium	1002	Seven Mile Beach NP	0.67 ± 0.36
**820a74cb34678c22bddf0be11d75e893**	d__Bacteria; p__Firmicutes; c__Clostridia; o__Lachnospirales; f__Lachnospiraceae	1000	Sharewater	0.49 ± 0.19
			Broulee	0.42 ± 0.11
			Murramarang NP	0.31 ± 0.07
			Monga NP	0.02 ± 0.02
**9f5d77c743e4e8fd92851d3fe40b323e**	d__Bacteria; p__Proteobacteria; c__Gammaproteobacteria; o__Burkholderiales	994	Broulee	0.21 ± 0.04
			Eurobodalla NP	0.12 ± 0.02
**01cf9a65286e6b93cc6c83aef2e7f19e**	d__Bacteria; p__Bacteroidota; c__Bacteroidia; o__Bacteroidales	988	Seven Mile Beach NP	1.05 ± 0.24
**d9a15c071f0df2e405f60ebbd8d5cb61**	d__Bacteria; p__Bacteroidota; c__Bacteroidia; o__Bacteroidales	982	Murramarang NP	0.41 ± 0.13
			Meroo NP	0.39 ± 0.03
			Broulee	0.21 ± 0.05
			Eurobodalla NP	0.07 ± 0.07

**Table A3:** KOs coding for plant-fibre-degrading genes across southern greater glider gut microbiomes from southeastern NSW. KEGG orthologue names and functional pathways taken from Kyoto Encyclopaedia of Genes and Genomes database [43]. Wilcoxon rank sum test used to test for statistically significant (*p* < 0.05) differences between groups. Mean relative abundance (%) ± standard error of mean (SEM) given to 2 significant figures. * = *p* < 0.05.

Fibre Degraded	KEGG Orthologue	Enzyme	Mean Relative Abundance (% ± SEM)		*p* Value
Cellulose	K01179	Endoglucanase	Broulee	0.077% ± 0.012%	0.10
			Eurobodalla NP	0.094% ± 0.016%	
			Meroo NP	0.053% ± 0.0050%	
			Monga NP	0.068% ± 0.0053%	
			Murramarang NP	0.072% ± 0.0078%	
			Seven Mile Beach NP	0.083% ± 0.0059%	
			Sharewater	0.048% ± 0.0096%	
	K05350	Beta-glucosidase	Broulee	0.028% ± 0.0026%	0.07
			Eurobodalla NP	0.037% ± 0.0041%	
			Meroo NP	0.033% ± 0.0010%	
			Monga NP	0.033% ± 0.0015%	
			Murramarang NP	0.036% ± 0.0023%	
			Seven Mile Beach NP	0.036% ± 0.0016%	
			Sharewater	0.020% ± 0.0022%	
Pectin	K18650	Exo-poly-alphagalacturonosidase	Broulee	0.00048% ± 0.000046%	0.03 *
			Eurobodalla NP	0.00093% ± 0.00029%	
			Meroo NP	0.00032% ± 0.000027%	
			Monga NP	0.00060% ± 0.00010%	
			Murramarang NP	0.00078% ± 0.000024%	
			Seven Mile Beach NP	0.00085% ± 0.000039%	
			Sharewater	0.00040% ± 0.0000052%	
	K06113	Arabinan endo-1,5-alpha-Larabinosidase	Broulee	0.021% ± 0.0029%	0.18
			Eurobodalla NP	0.025% ± 0.0048%	
			Meroo NP	0.024% ± 0.00057%	
			Monga NP	0.024% ± 0.0020%	
			Murramarang NP	0.030% ± 0.0025%	
			Seven Mile Beach NP	0.027% ± 0.0029%	
			Sharewater	0.017% ± 0.0017%	
	K01051	Pectinesterase	Broulee	0.0040% ± 0.00053%	0.10
			Eurobodalla NP	0.0029% ± 0.00081%	
			Meroo NP	0.0034% ± 0.00097%	
			Monga NP	0.0035% ± 0.00083%	
			Murramarang NP	0.0033% ± 0.0017%	
			Seven Mile Beach NP	0.0013% ± 0.000091%	
			Sharewater	0.0019% ± 0.00071%	
	K01728	Pectate lyase	Broulee	0.00020% ± 0.000088%	0.25
			Eurobodalla NP	0.00011% ± 0.00011%	
			Meroo NP	0.00016% ± 0.000064%	
			Monga NP	0.00024% ± 0.000098%	
			Murramarang NP	0.00017% ± 0.000078%	
			Seven Mile Beach NP	0.000075% ± 0.000027%	
			Sharewater	-	
Xylan	K01181	Endo-1,4-beta-xylanase	Broulee	0.018% ± 0.0031%	0.04 *
			Eurobodalla NP	0.015% ± 0.0039%	
			Meroo NP	0.0053% ± 0.00049%	
			Monga NP	0.011% ± 0.0011%	
			Murramarang NP	0.012% ± 0.0038%	
			Seven Mile Beach NP	0.015% ± 0.0016%	
			Sharewater	0.0079% ± 0.0021%	
	K15924	Glucuronoarabinoxylan endo-1,4-beta-xylanase	Broulee	0.0065% ± 0.0016%	0.04 *
			Eurobodalla NP	0.0032% ± 0.0021%	
			Meroo NP	0.0013% ± 0.00040%	
			Monga NP	0.0025% ± 0.00049%	
			Murramarang NP	0.0045% ± 0.0015%	
			Seven Mile Beach NP	0.0066% ± 0.00074%	
			Sharewater	0.0037% ± 0.0014%	
	K01811	Alpha-D-xyloside xylohydrolase	Broulee	0.025% ± 0.0027%	0.03 *
			Eurobodalla NP	0.017% ± 0.0027%	
			Meroo NP	0.015% ± 0.0019%	
			Monga NP	0.021% ± 0.0021%	
			Murramarang NP	0.022% ± 0.0017%	
			Seven Mile Beach NP	0.025% ± 0.00057%	
			Sharewater	0.012% ± 0.000057%	
	K01805	Xylose isomerase	Broulee	0.017% ± 0.0026%	0.06
			Eurobodalla NP	0.0053% ± 0.0024%	
			Meroo NP	0.016% ± 0.0027%	
			Monga NP	0.012% ± 0.0021%	
			Murramarang NP	0.014% ± 0.0040%	
			Seven Mile Beach NP	0.016% ± 0.0013%	
			Sharewater	0.0063% ± 0.00012%	

**Table A4:** KOs coding for plant-fibre-degrading genes across southern greater glider gut microbiomes from burnt and unburnt sites. Wilcoxon rank sum test used to test for statistically significant (*p* < 0.05) differences between groups. Mean relative abundance (%) ± standard error of mean (SEM) given to 2 significant figures. Test statistic (S) and *p* values indicated. * = *p* < 0.05.

Fibre Degraded	KEGG Orthologue	Enzyme	Mean Relative Abundance (% ± SEM)		S	*p* Value
			Burnt Habitat	Unburnt Habitat		
Cellulose	K01179	Endoglucanase	0.063% ± 0.0039%	0.083% ± 0.0062%	190	0.01 *
	K05350	Beta-glucosidase	0.032% ± 0.0016%	0.033% ± 0.0019%	146	0.89
Pectin	K18650	Exo-poly-alphagalacturonosidase	0.00055% ± 0.000062%	0.00072% ± 0.000097%	172	0.12
	K06113	Arabinan endo-1,5-alpha-Larabinosidase	0.024% ± 0.0014%	0.024% ± 0.0020%	141	0.93
	K01051	Pectinesterase	0.0032% ± 0.00051%	0.0027% ± 0.00044%	131	0.53
	K01728	Pectate lyase	0.00017% ± 0.000049%	0.00013% ± 0.000044%	131	0.53
Xylan	K01181	Endo-1,4-beta-xylanase	0.0095% ± 0.0011%	0.016% ± 0.0015%	195	<0.01 *
	K15924	Glucuronoarabinoxylan endo-1,4-beta-xylanase	0.0028% ± 0.00048%	0.0057% ± 0.00089%	191	<0.01 *
	K01811	Alpha-D-xyloside xylohydrolase	0.018% ± 0.0014%	0.023% ± 0.0016%	178	0.59
	K01805	Xylose isomerase	0.012% ± 0.0014%	0.013% ± 0.0064%	157	0.46

**Table A5:** Differentially abundant KOs detected between southern greater glider gut microbiomes from across southeastern NSW. ANCOM used to determine significantly differentially abundant KOs between locations. W value represents the number of times the null hypothesis (the average abundance of a KO in one population is equal to that in another population) was rejected for a given KO, under a 0.05 significance threshold. Only significantly differentially abundant KOs are listed. KEGG orthologue names and functional pathways taken from Kyoto Encyclopaedia of Genes and Genomes database [43]. Relative abundance given as mean (%) ± standard error of the mean (SEM) for locations where the respective KO was detected in at least one sample.

KO	Name	Pathways	W	Mean Relative Abundance (% ± SEM)	
K08256	Phosphatidyl-myo-inositol alpha-mannosyltransferase	Lipoarabinomannan biosynthesis Metabolic pathways	4388	Meroo NP	0.00015 ± 0.00007
				Eurobodalla NP	0.00015 ± 0.000001
				Murramarang NP	0.00011 ± 0.00044
				Broulee	0.00010 ± 0.00003
				Seven Mile Beach NP	0.00003 ± 0.00001
				Monga NP	<0.00001%
K11779	F0 synthase	Methane metabolism Metabolic pathways Microbial metabolism in diverse environments	3894	Meroo NP	0.00016 ± 0.00008
				Eurobodalla NP	0.00015 ± 0.000002
				Murramarang NP	0.00011 ± 0.00004
				Broulee	0.00010 ± 0.00003
				Seven Mile Beach NP	0.00003 ± 0.00001
K11263	Acetyl-CoA/propionyl-CoA carboxylase, biotin carboxylase, biotin carboxyl carrier protein	Fatty acid biosynthesis Valine, leucine and isoleucine degradation Pyruvate metabolism Glyoxylate and dicarboxylate metabolism Propanoate metabolism Metabolic pathways Biosynthesis of secondary metabolites Microbial metabolism in diverse environments Carbon metabolism Fatty acid metabolism	3863	Meroo NP	0.00031 ± 0.00021
				Eurobodalla NP	0.00017 ± 0.00002
				Murramarang NP	0.00011 ± 0.00005
				Broulee	0.00011 ± 0.00004
				Seven Mile Beach NP	0.00003 ± 0.00001
				Monga NP	<0.00001
